# Delayed-Type Hypersensitivity: An Excellent Indicator of Anti-tumor Immunity With Wilms' Tumor 1 (WT1) Dendritic Cell Vaccine Therapy

**DOI:** 10.7759/cureus.49221

**Published:** 2023-11-22

**Authors:** Hisashi Nagai, Ryusuke Karube

**Affiliations:** 1 Human and Environmental Studies, Tokai University, Hiratsuka, JPN; 2 Oncology, Ginza Phoenix Clinic, Tokyo, JPN

**Keywords:** advanced cancer, immune profile, wt1, cancer immunotherapy, dendritic cell vaccine

## Abstract

Introduction: It is a well-known fact that anti-tumor immunity is a crucial long-term survival factor in cancer. Wilms' tumor 1 (WT1) dendritic cell vaccine therapy (WT1-DC) is an immuno-cell therapy that has been implemented against various cancers as a tumor-specific immunotherapy targeting the common cancer antigen WT1.

Methods: Seven doses of WT1-DC vaccine were administered to six patients, three of whom had stage IV lung cancer with metastases and the other three had stage IV pancreatic cancer with metastases, all of whom were receiving chemotherapy and had similar physical conditions. Their immune response was assessed using delayed-type hypersensitivity (DTH) and immune profile status (IPS) such as blood neutrophil percentage, lymphocyte percentage, and neutrophil-to-lymphocyte (N/L) ratio.

Results: In lung cancer, DTH increased with repeated DC administration, and IPS improved with it, whereas in pancreatic cancer, DTH did not increase, and IPS worsened from the fifth inoculation. Fever in the 37° range was observed after DC administration in lung cancer, but not in pancreatic cancer.

Conclusion: These results suggest that DTH and IPS are correlated in dynamics and that DTH is a good indicator of the state of anti-tumor immunity. Since IPS is a prognostic factor in advanced cancer, the magnitude of DTH due to WT1-DC inoculation is a useful indicator to estimate the patient's prognosis. Although DTH is an extremely simple test, its clinical significance has not been fully investigated. The present study demonstrates the importance of DTH in cancer treatment with WT1-DC.

## Introduction

In recent years, anti-tumor immunity has become an important aspect of cancer treatment. Clinical studies with immune checkpoint inhibitors have shown that enhanced anti-tumor immunity significantly prolongs overall survival (OS) [1,2].

In clinical studies of cell-based therapies for cancer conducted around the world, antigen-presenting cell-based therapies are the second most commonly performed after chimeric antigen receptor (CAR)-T cell therapy [3]. Dendritic cells are commonly used as antigen-presenting cells, and the common cancer antigen Wilms' tumor 1 (WT1) is often used as their target antigen.

According to a 2009 report by the National Cancer Institute in the USA, the WT1 antigen ranked first among 75 common cancer antigens in an overall score of nine categories, including therapeutic efficacy, immunogenicity, cancer specificity, and expression [4]. Lung and pancreatic cancers express WT1 more than 90% of the time, making WT1 a good immune target [5].

The induction of WT1-specific cytotoxic T lymphocytes (WT1-CTL) by WT1 dendritic cell vaccine therapies (WT1-DCs) can be assessed by delayed-type hypersensitivity (DTH) by intradermal administration of the WT1 antigen [6-8]. On the other hand, the number and ratio of immune cells by blood tests or blood levels of inflammatory substances such as C-reactive protein (CRP) are known as prognostic factors in cancer [9-13]. We call these indicators "immune profile status" (IPS) and have already reported their clinical significance [14]. However, no study has investigated the correlation between DTH and IPS.

We, therefore, compared DTH and IPS by WT1-DC in a group of stage IV lung and pancreatic cancer patients in similar physical conditions. Compared to lung cancer, pancreatic cancer is known to have lower immunogenicity and a lower response rate to immunotherapy. Therefore, the correlation between DTH and IPS can be determined by examining the immune response of WT1-DCs in lung and pancreatic cancers. Here, the significance of DTH in WT1-DC is discussed based on research results and previous literature.

## Materials and methods

Patient

The ethics committee of the Institutional Review Board at Ginza Phoenix Clinic reviewed and approved this study (approval number: 23-01). WT1 dendritic cell vaccine therapy was administered to three patients with stage IV lung cancer with metastases and three patients with stage IV pancreatic cancer with metastases. The types of lung cancer were adenocarcinoma in two patients and squamous cell carcinoma in one patient. All pancreatic cancers were adenocarcinomas. Their ages ranged from 60 to 70 years. Apart from one female in the pancreatic cancer group, all are males. None of the patients had a disease other than cancer. All were undergoing multi-agent chemotherapy but were physically fit with a performance status (PS) of 1-2. PS is one of the indicators of general condition and indicates the degree of limitation of the patient's daily activities. PS1 is a condition that limits physically strenuous activities, but the patient is able to walk and perform light or sedentary tasks. PS2 means that the patient is ambulatory and able to do all their own personal activities, but not work. PS2 is a condition in which the patient spends more than 50% of the day outside the bed.

DC preparation

Peripheral blood mononuclear cells (PBMCs) were prepared from white blood cell collections by Ficoll high-pack gradient density centrifugation. PBMCs were then plated in tissue culture vessels and cultured continuously in a medium containing granulocyte-monocyte colony-stimulating factor (50 ng/mL) and interleukin-4 (25 ng/mL) for five days to generate immature DCs. To further induce differentiation, immature DCs were stimulated with OK-432 (Chugai Pharmaceutical Co., Tokyo, Japan) and prostaglandin-E2 (50 ng/mL) (Daiichi Fine Chemical Co., Toyama, Japan) for 24 hours. On day 7 of culture, DCs were pulsed with WT1 peptide antigen. DCs were then characterized by flow cytometry to ensure that they had reached the typical phenotype of mature DCs (CD14-/low/HLA-DR+/HLA-ABC+/HLA-ABC+/CD80+/CD83+/CD86+/CD40+/CCR7+). They were then frozen and stored until the day of administration.

Treatment

The DC suspension was adjusted to a total volume of 1 mL using saline. All patients received a total of seven subcutaneous injections of WT1-DC near the right and left inguinal lymph nodes. Injections were repeated every 14 days. As all patients were free of a severe allergy to penicillin or other drugs, the streptococcal immunological adjuvant OK-432 was administered simultaneously with the DC vaccine; the tolerated dose of OK-432 ranged from 1 to 5 KE, and the appropriate dose was determined by the incidence of fever following administration.

Assessments of immune response

**Figure 1:**
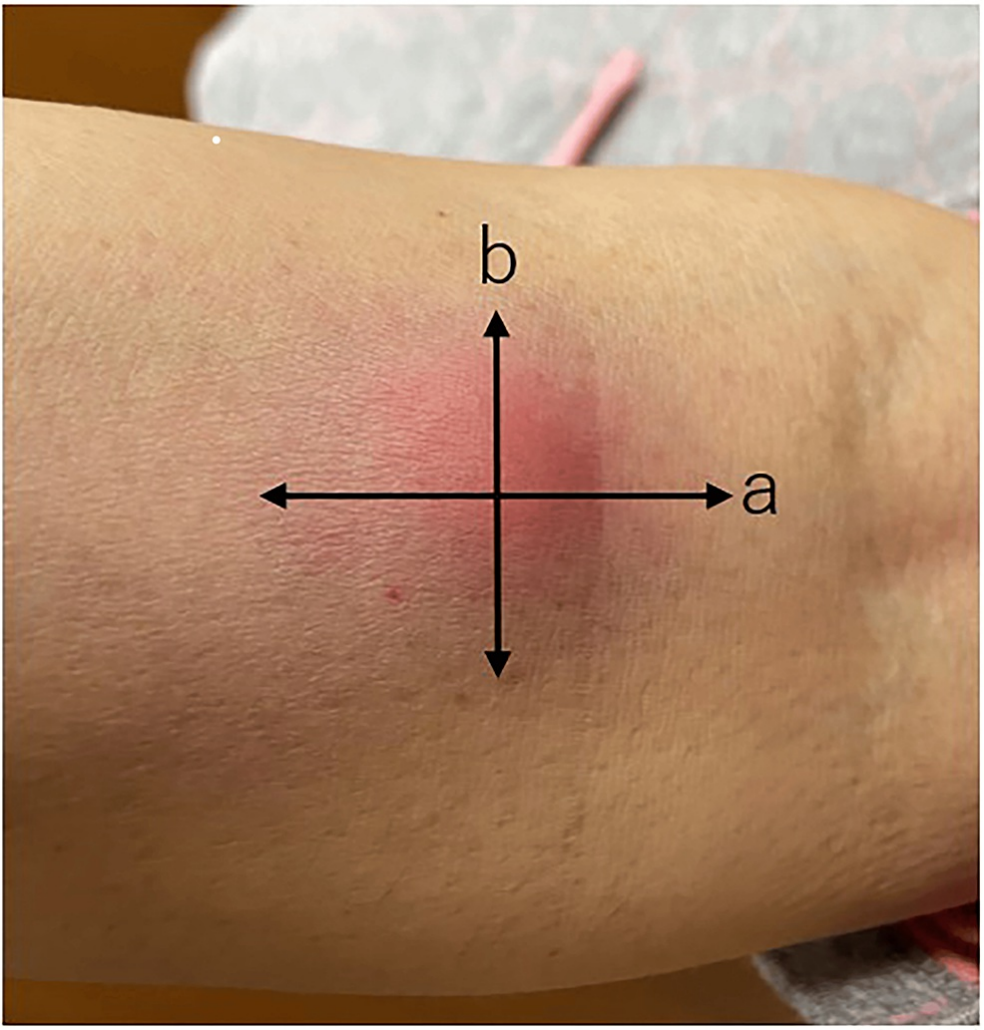
Delayed-type hypersensitivity The size of the erythema is measured within three days after the administration of WT1 antigen to the anterior forearm. The longest (a) and shortest (b) diameters are measured from the point where the erythema disappears completely, and the mean value is calculated.

At the time of WT1-DC administration, 0.1 mL of WT1 antigen was injected intradermally into the medial forearm, and DTH was measured. The long and short diameters of transient erythema were plural times measured in patients within 72 hours after each vaccination, and the mean value was calculated (Figure 1). Post-vaccination fever was also assessed, and the maximum body temperature (BT max) within 72 hours after vaccination was noted. In addition, the number of white blood cells in the blood, neutrophil percentage, lymphocyte percentage, neutrophil-to-lymphocyte (N/L) ratio, and CRP levels were measured as IPS, an indicator of systemic immune response.

Evaluation

All adverse events were assessed and recorded according to Common Terminology Criteria for Adverse Events version 6.0. Clinical assessments were performed by CT or MRI at baseline and at 1-2 months after the completion of WT1-DC. Response rates were expressed as complete response (CR), partial response (PR), stable disease (SD), or disease progression (PD) according to Response Evaluation Criteria in Solid Tumours (RECIST) version 1.1.

Statistical analysis

A comparison of the results of blood sampling at the first dose of WT1-DC was statistically analyzed using a t-test. DTH, BT max, and hematologic parameters were statistically analyzed with multiple comparisons for two-way analysis of variance (ANOVA). Probability (P) values of less than 0.05 were two-sided and considered statistically significant.

## Results

Age was 65.7±2.9 years in the lung cancer group and 70.67±9.2 years in the pancreatic cancer group, with no significant difference between the two groups. Both were undergoing multi-agent chemotherapy, but at the start of WT1-DC, they had only grade 1 side effects such as mild malaise and a decrease in appetite and performance status of 1 or 2. Body mass index (BMI) was 23.1±2.8 in the lung cancer group and 20.8±2.0 in the pancreatic cancer group, with no significant difference between the two groups (Table 1).

**Table 1:** Patient characteristics Performance status, chemotherapy side effects, and BMI indicate status on the date of the first dose of WT1-DC. The Time when WT1-DC was started and Outcome indicate the number of days from the date of diagnosis. M: male, F: female, BMI: body mass index, PR: partial response, PD: progressive disease, SD: stable disease, WT1-DC: WT1 dendritic cell vaccine therapy

	Lung cancer			Pancreatic cancer		
Age	64	69	64	76	60	70
Sex	M	M	M	F	M	M
Stage	Ⅳ	Ⅳ	Ⅳ	Ⅳ	Ⅳ	Ⅳ
Metastasis	Brain	Liver, bone, lung, adrenal gland	Lung	Liver, peritoneum	Liver	Liver, kidney, adrenal gland
Chemotherapy	Carboplatin, pemetrexed, pembrolizumab	Docetaxel, ramucirumab	Carboplatin, pemetrexed	Gemcitabine, nab-paclitaxel	Gemcitabine, tegafur/gimeracil/oteracil	Nimotuzumab, tegafur/gimeracil/oteracil
Performance status	1	2	1	2	1	2
Side effects of chemotherapy	Decrease in appetite, mild malaise	Mild malaise	Mild malaise	Mild malaise, mild edema	None	Decrease in appetite, decline in physical strength
BMI	26.1	22.5	20.6	23.1	19.6	19.8
Time when WT1-DC was started	Day 119	Day 200	Day 201	Day 84	Day 112	Day 496
Assessment after WT1-DC	PR	PR	PR	PD	SD	SD
Outcome	Alive, 364 days	Alive, 637 days	Alive, 668 days	Died, 144 days	Died, 425 days	Died, 790 days

Blood tests at the time of the first WT1-DC administration showed no difference in white blood cell count, red blood cell count, hemoglobin concentration, or platelet count. Overall values were slightly lower, although not obviously pancytopenic. Total protein and albumin levels were normal in both groups, and there was no obvious malnutrition. In the pancreatic cancer group, γ-glutamyl transpeptidase (γGTP) was slightly elevated, but bilirubin levels were not abnormal. Blood creatinine levels were slightly higher in the lung cancer group with a significant difference (Table 2).

**Table 2:** Blood test findings Blood test findings on the day of the first WT1-DC administration. Values are expressed as mean±SD. *p<0.05 WBC: white blood cell, RBC: red blood cell, Hb: hemoglobin, Plt: platelet, T-bil: total bilirubin, D-bil: direct bilirubin, TP: total protein, Alb: albumin, AST: aspartate aminotransferase, ALT: alanine aminotransferase, γGTP: γ-glutamyl transpeptidase, Cre: creatinine, SD: standard deviation

	Lung cancer	Pancreatic cancer	P value
WBC (/uL)	6300±1087	6256±2713	0.9807
RBC (×10^4^/uL)	395±24	348±41	0.1601
Hb (g/dL)	11.8±1.0	11.4±0.4	0.4750
Plt (×10^4^/uL)	19.9±7.5	18.3±0.4	0.7358
TP (g/dL)	6.9±0.8	6.5±0.0	0.3777
Alb (g/dL)	4.0±0.3	3.7±0.4	0.2794
T-bil (mg/dL)	0.4±0.1	0.5±0.2	0.1518
D-bil (mg/dL)	0.1±0.0	0.2±0.1	0.1161
AST (U/L)	31.7±22.7	24.7±7.8	0.6402
ALT (U/L)	31.7±19.4	16.7±10.8	0.3068
γGTP (U/L)	43.7±14.6	109.3±35.2	0.0406*
Cre (mg/dL)	1.1±0.2	0.7±0.1	0.0279*

**Figure 2:**
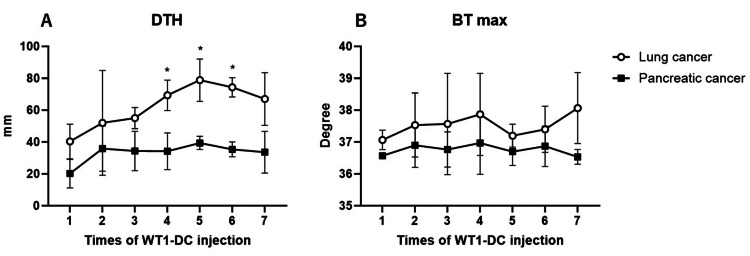
Changes in DTH and body temperature A: Mean DTH within 72 hours after WT1-DC administration. In the lung cancer group, DTH increases as the number of WT1-DC doses increases, whereas in the pancreatic cancer group, it does not increase after the second dose. B: Maximum body temperature within 72 hours after WT1-DC administration. Although not significantly different, the lung cancer group had a higher body temperature after WT1-DC administration than the pancreatic cancer group. The pancreatic cancer group does not have a fever. Values are expressed as mean±SD. *p<0.05 DTH: delayed-type hypersensitivity, WT1-DC: WT1 dendritic cell vaccine therapy, BT max: maximum body temperature, SD: standard deviation

The number of cells administered per dose of WT1-DC was 22.47±2.20x10^6^ in the lung cancer group and 25.13±4.15x10^6^ in the pancreatic cancer group, with no significant differences between the two groups. DTH was greater in the lung cancer group than in the pancreatic cancer group for all WT1-DC inoculations; there were statistically significant differences for the fourth, fifth, and sixth DTH. The average size of DTH in the lung cancer group was 40 mm in diameter at the first dose and more than 60 mm at the third through seventh doses. The pancreatic cancer group was 20 mm at the first dose and remained below 40 mm throughout all doses. The body temperature of the lung cancer group averaged 37.1°C at the first administration and remained above 37°C thereafter. On the other hand, the temperature in the pancreatic cancer group was in the 36°C range at all times except for the fourth dose (37°C) (Figure 2).

**Figure 3:**
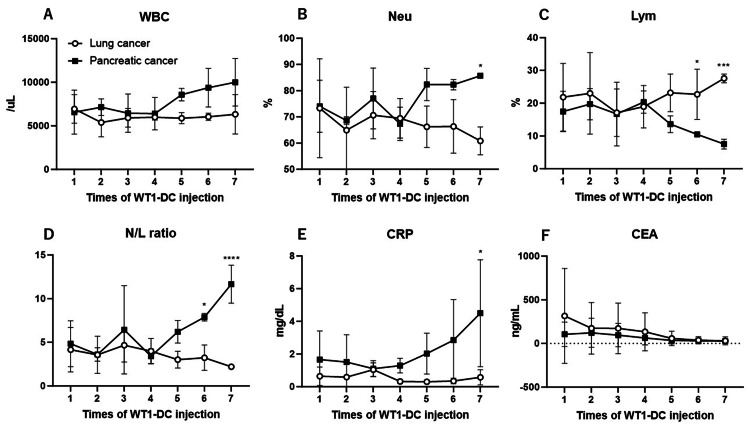
Hematologic parameters Variation in hematologic parameters during post-administration of WT1-DC. A: White blood cell, B: neutrophil, C: lymphocyte, D: neutrophil-to-lymphocyte ratio, E: CRP, F: CEA Values are expressed as mean±SD. *p<0.05, *** p<0.001, ****p<0.0001 WBC: white blood cell, Neu: neutrophil, Lym: lymphocyte, N/L: neutrophil/lymphocyte, CRP: C-reactive protein, CEA: carcinoembryonic antigen, SD: standard deviation

In blood tests, the lung cancer group showed no change in the total white blood cell count, but the neutrophil percentage decreased, the lymphocyte percentage increased, the N/L ratio decreased, and the CRP level decreased mildly. In the pancreatic cancer group, the total white blood cell counts increased, the neutrophil percentage increased, the lymphocyte percentage decreased, and the N/L ratio and CRP levels increased rapidly. These changes were observed as obvious changes from the fifth WT1-DC (Figure 3). Carcinoembryonic antigen (CEA), a common tumor marker in lung and pancreatic cancer, decreased in both groups. Carbohydrate antigen 19-9 (CA19-9), duke pancreatic monoclonal antigen type 2 (DU-PAN-2), and s-pancreas antigen-1 (SPan-1) were also measured in pancreatic cancer, but no consistent trend was observed (data not shown). In all patients, WT1-DC inoculation did not cause any side effects other than DTH and fever.

One to two months after the completion of WT1-DC treatment, the size of the cancers was assessed by CT and MRI scans, and all patients in the lung cancer group had PR, while two patients in the pancreatic cancer group had SD, and one patient had PD. At the time of writing this paper, there were no deaths in the lung cancer group, but in the pancreatic cancer group, all died due to exacerbation of pancreatic cancer (Figure 1).

## Discussion

This paper compares the results of seven doses of WT1-DC inoculation in three patients each with stage IV lung and pancreatic cancer with metastases. The physical status of the patients in both groups was quite close at the beginning of treatment in terms of concomitant chemotherapy, degree of chemotherapy side effects, PS, BMI, and other key blood test parameters. γGTP was slightly higher in the pancreatic cancer group, but this may be due to the fact that all patients with pancreatic cancer had liver metastases. Blood creatinine levels were slightly higher in the lung cancer group, but the cause was unknown. None of the patients had symptoms of hepatic or renal dysfunction, and it was concluded that these differences in laboratory values had no effect on the therapeutic effect of WT1-DC.

As known from studies of immune checkpoint inhibitors, overall survival (OS) is significantly prolonged in groups of patients with enhanced anti-tumor immunity [1,2]. Therefore, anti-tumor immunity is extremely important in cancer therapy. An increase in the number of white blood cells in the blood, an increase in the percentage of neutrophils, a decrease in the percentage of lymphocytes, an increase in the N/L ratio, and an increase in CRP levels are known as poor prognostic factors for advanced cancer [9-13]. We call these immune-related indicators "immune profile status" and have reported that WT1-DC therapy improves IPS and may contribute to prolonged progression-free survival [14]. On the other hand, DTH has been reported to be a useful indicator of WT1-CTL induction by WT1-DC in previous reports [6-8]. However, no study has examined the relationship between DTH and IPS over time in response to the number of times WT1-DC is administered.

Late-stage pancreatic cancer has an extremely poor prognosis among cancers, with a five-year survival rate of 2% [15]. Pancreatic cancer is known for its rapid growth rate and difficulty in responding to immunotherapy such as immune checkpoint inhibitors. Therefore, immune checkpoint inhibitors are usually not used for pancreatic cancer. In contrast, non-small cell lung cancer is treated with immune checkpoint inhibitors even at stage IV. Overall, lung cancer is more likely to activate anti-tumor immunity than pancreatic cancer [15,16]. Based on these characteristics, we compared DTH and IPS of WT1-DC treatment in patients with end-stage lung and pancreatic cancer with similar physical conditions.

The results of this study show that DTH was always larger in the lung cancer group than in the pancreatic cancer group, and in the lung cancer group, DTH tended to increase as the number of times WT1-DC was administered increased. Conversely, the DTH in the pancreatic cancer group did not increase much with the number of doses and was always less than 40 mm. There were differences in the magnitude of DTH in the fourth, fifth, and sixth doses that were statistically significant, even among the small number of groups (n=3). Body temperature after DC administration was also higher in the lung cancer group than in the pancreatic cancer group, with temperatures above 37°C seen in the lung cancer group, but not in the pancreatic cancer group.

Although larger DTH has been reported to be more clinically effective against cancer, there is no unified view on the numerical value of erythema diameter and its clinical efficacy. Some reports have used a diameter of 30 mm as a cutoff and found significant differences, while others have used a diameter of 5 mm. Others do not state the size of the diameter but classify only by the presence or absence of DTH [6-8]. One possible cause of this may be differences in the way DTH is measured. The values vary greatly depending on where the erythematous end of the DTH is set. In our clinic, we have standardized the method of measuring the outermost part of the erythema's pale margin, which can be recognized by the naked eye, as the edge of the erythema (Figure 1). Although the types of antigens and culture methods used in dendritic cell vaccine therapy vary from report to report, the method of measuring DTH must be defined for comparative studies of its effectiveness. We believe that a uniform method should be adopted in which the outermost point of the visible erythema is the starting point for measurement and the average of the long and short diameters is taken as the DTH.

A key feature of the results of the present study is that DTH and IPS are linked in time. During the WT1-DC treatment period, IPS improves with increasing DTH in the lung cancer group, while IPS worsens in the pancreatic cancer group, which is consistent with the lack of DTH increase. Moreover, after the size of DTH in the lung cancer group increases rapidly to over 60 mm in diameter at the fourth DC inoculation, the IPS value enters an improving trend at the fifth DC inoculation. Since WT1-DC was inoculated every two weeks, the increase in DTH may precede the improvement in IPS by at least two weeks. Conversely, in the pancreatic cancer group, DTH changed little after the second inoculation, and IPS worsened rather from the fifth inoculation.

The absence of an increase in DTH and the weaker induction of an effective WT1-specific cellular immune response compared to the lung cancer group indicates that anti-tumor immunity is strongly suppressed in pancreatic cancer, which is consistent with previous knowledge [15,17,18]. On the other hand, there are reports of prolonged OS in pancreatic cancer patients when WT1-DC is combined with multi-agent chemotherapy [6]. It has also been shown that WT1-CTLs are induced by WT-DC administration in pancreatic cancer patients undergoing chemotherapy, and a higher number of CTLs is associated with a longer OS [19,20]. The degree of immunosuppression in pancreatic cancer varies from patient to patient. Depending on the combination with standard treatment including chemotherapy, WT1-DC may enhance anti-tumor immunity. In cancers other than pancreatic cancer, it is presumed that WT1-DC does not increase DTH in cancer patients with strongly suppressed antitumor immunity. Changes in the magnitude of DTH are not only suggestive of changes in IPS but also serve as a useful prognostic indicator in that cancer patient.

## Conclusions

Here, we report that DTH indicates the status of IPS and can be an indicator of the patient's prognosis. In cases where DTH values are continuously increased with WT1-DC therapy, it is thought that the activation of anti-tumor immunity is occurring along with the improvement of IPS, increasing the possibility of prolonged overall survival even in late-stage cancer. Therefore, the evaluation of DTH in WT1-DC therapy is important, and precise measurement is essential. This is why the DTH measurement method needs to be standardized.

In this study, it is unclear through what mechanism the induction of cytotoxic T cells targeting the WT1 antigen elicited by WT1-DC affects the anti-tumor immunity of the body as a whole. Future studies are needed to determine the impact of immuno-cell therapy targeting specific cancer antigens on systemic immunity.
